# Managing clustering effects and learning effects in the design and analysis of randomised surgical trials: a review of existing guidance

**DOI:** 10.1186/s13063-022-06743-6

**Published:** 2022-10-11

**Authors:** Elizabeth J. Conroy, Jane M. Blazeby, Girvan Burnside, Jonathan A. Cook, Carrol Gamble

**Affiliations:** 1 Liverpool Clinical Trials Centre, University of Liverpool, Liverpool, UK; 2 Oxford Clinical Trials Research Unit, Centre for Statistics in Medicine, Nuffield Department of Orthopaedics, Rheumatology and Musculoskeletal Sciences, University of Oxford, Windmill Road, Oxford, OX3 7LD UK; 3 Centre for Surgical Research, Bristol Biomedical Research Centre, Population Health Sciences, University of Bristol, Bristol, UK

**Keywords:** Trials, Clinical trial, Randomised controlled trial, Complex intervention, Surgical intervention, Trial design, Trial analysis, Summary, Review, Clustering, Learning

## Abstract

**Background:**

The complexities associated with delivering randomised surgical trials, such as clustering effects, by centre or surgeon, and surgical learning, are well known. Despite this, approaches used to manage these complexities, and opinions on these, vary. Guidance documents have been developed to support clinical trial design and reporting. This work aimed to identify and examine existing guidance and consider its relevance to clustering effects and learning curves within surgical trials.

**Methods:**

A review of existing guidelines, developed to inform the design and analysis of randomised controlled trials, is undertaken. Guidelines were identified using an electronic search, within the Equator Network, and by a targeted search of those endorsed by leading UK funding bodies, regulators, and medical journals. Eligible documents were compared against pre-specified key criteria to identify gaps or inconsistencies in recommendations.

**Results:**

Twenty-eight documents were eligible (12 Equator Network; 16 targeted search). Twice the number of guidance documents targeted design (*n*/*N*=20/28, 71%) than analysis (*n*/*N*=10/28, 36%). Managing clustering by centre through design was well documented. Clustering by surgeon had less coverage and contained some inconsistencies. Managing the surgical learning curve, or changes in delivery over time, through design was contained within several documents (*n*/*N*=8/28, 29%), of which one provided guidance on reporting this and restricted to early phase studies only. Methods to analyse clustering effects and learning were provided in five and four documents respectively (*N*=28).

**Conclusions:**

To our knowledge, this is the first review as to the extent to which existing guidance for designing and analysing randomised surgical trials covers the management of clustering, by centre or surgeon, and the surgical learning curve. Twice the number of identified documents targeted design aspects than analysis. Most notably, no single document exists for use when designing these studies, which may lead to inconsistencies in practice. The development of a single document, with agreed principles to guide trial design and analysis across a range of realistic clinical scenarios, is needed.

**Supplementary Information:**

The online version contains supplementary material available at 10.1186/s13063-022-06743-6.

## Background

Randomised controlled trials (RCTs) are recognised as providing the highest level of evidence, second only to systematic reviews of such trials [1]. There are many practical and methodological difficulties that a medical researcher must overcome to deliver successful RCT. In trials with a surgical intervention, these difficulties are often magnified [2–5]. Surgical interventions, delivered as an intervention or as a setting, consist of many interacting components — such as the procedure itself, surgeon expertise, and pre- or postoperative care [4].

Patient outcomes often depend on the treatment provider delivering the intervention. Due to the nature of surgical interventions, RCTs within this field can be vulnerable to criticism if concerns over variability in treatment delivery are raised. Variability can arise between intervention providers (clustering) or due to change in delivery over time, often as a result of increased experience (learning) [6–8]. Therefore, when designing these trials, it is important to consider the homogeneity of the treatment effect and therefore the potential existence and impact of both clustering and learning, by centre and surgeon. This should be done as early as possible during trial design to avoid issues arising that violate the validity of the trial results [9].

The importance of managing these effects within these trials is well known, but the methods used to do so in practice, and opinions on these, vary [10–12]. Guidance documents exist to support clinical trial design and reporting, but the majority target generic aspects of clinical trials and originate specifically from medicinal trials. Therefore, whilst their relevance to all trials is indisputable, the extent to which they cover clustering and learning may be limited.

The aim of this review is to identify and examine existing guidance and consider its relevance to clustering effects and learning curves within surgical trials.

## Methods

This work sought to include guidance documents developed to inform the design and analysis of randomised controlled trials (RCTs). Guidelines for inclusion in this review were identified by undertaking:

- An electronic search within the Equator Network (http://www.equator-network.org), an online library containing a comprehensive searchable database of reporting guidelines, using each of the search terms ‘surgery’ and ‘statistic’. Documents that provided guidance specific to non-randomised studies, aspects of trial methodology or medical specialties that were not applicable, or focussed on applicable medical specialties, such as surgery, with no statistical scope were excluded.
- A targeted search of guidelines endorsed by leading UK funding bodies, regulators, and medical journals such that they covered aspects of trial design, analysis, and reporting.

Because of the nature of the search, full texts of identified guidelines were obtained to determine eligibility. Documents that provided guidance such that RCTs and statistical aspects were covered within their scope were included and reasons for exclusion were recorded.

Key criteria relevant to the design and analysis of surgical trials, or trials of complex interventions, were identified a priori (see Table 1). Eligible documents were compared against these to identify gaps or inconsistencies in recommendations. Guidelines for reporting the aspects of design and analysis were also assessed against these criteria. Specific methods within the guidelines related to analysing clustering or learning, at the centre or treatment provider level, were also collected. Documents were examined using NVivo qualitative data analysis software [13].

**Table 1:** Key criteria to be considered within design and analysis

**Design** • The appropriate trial design, such as an expertise-based design • Delivery of the intervention in terms of: ◦ The health professionals delivering treatment ◦ The extent to which treatments are to be standardised ◦ The potential for change in delivery over time • Adjusting the sample size • Balancing treatment within centres and treatment providers **Analysis** • When the randomisation has been stratified • When analysing the primary outcome, such as adjustment • When there are multiple centres and/or treatment providers	

## Results

### Identifying eligible documents

**Fig. 1:**
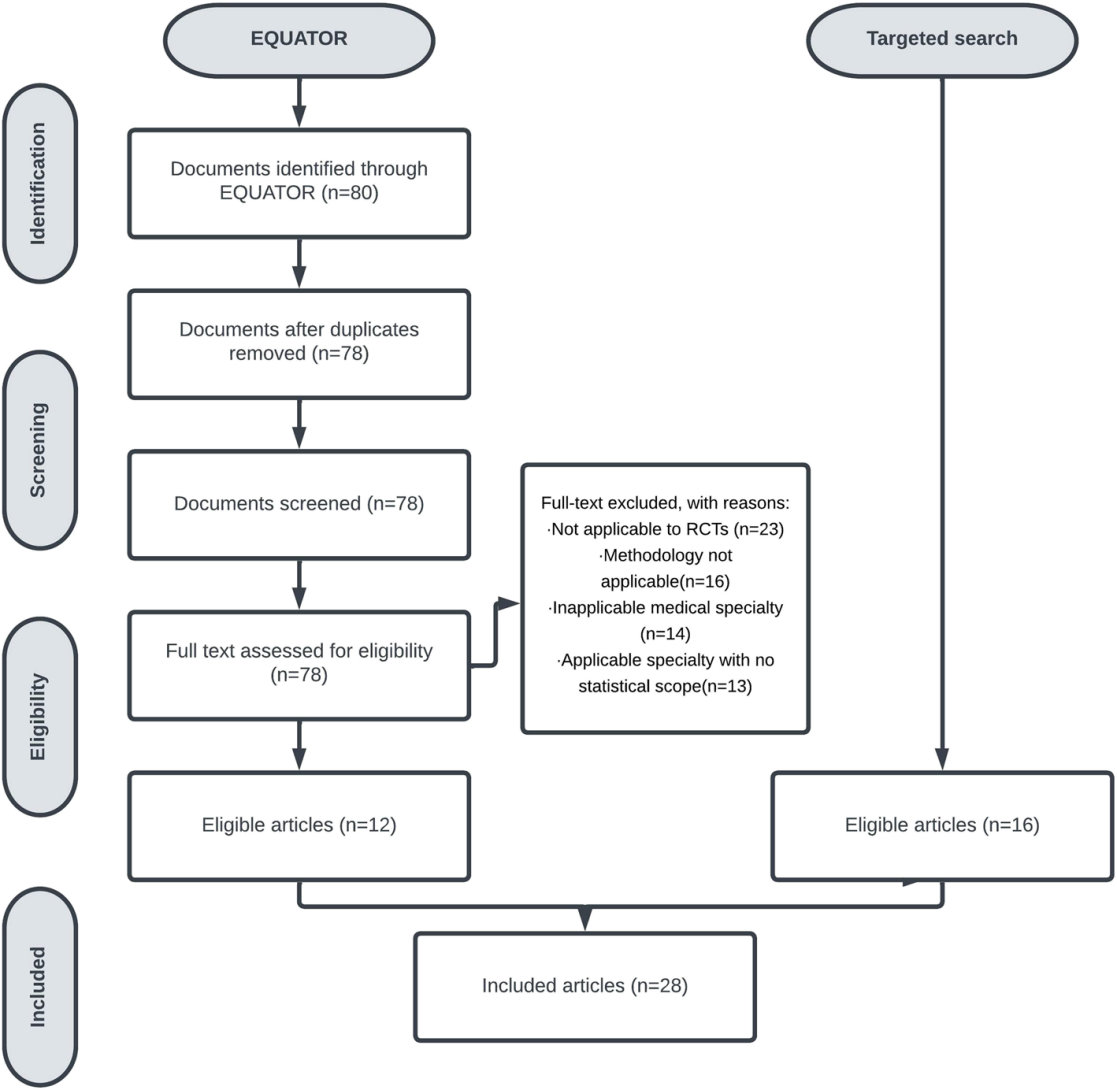
Flowchart of identification of guidelines

The search within the EQUATOR website identified 80 documents: 36 (45%) were identified using the search term ‘statistic’ and 44 (55%) using the search term ‘surgery’. The search was conducted on 21 October 2021. Figure 1 presents the flowchart of eligibility, with reasons for exclusion where necessary. An additional 16 documents were manually identified from the targeted search (funders: 2; regulators: 6; journals: 8, see Supplementary Material 1 and Fig. 1). There were no duplicates between the two searches leaving a total of 28 eligible documents for review. Supplementary Material 1 provides the list of included documents. Details as to the background and justification for the documents included as part of the targeted search are provided in Supplementary Material 2.

### Summary of identified guidance

Seven of the 28 eligible documents (25%) were developed specifically for surgery. Of those that were not (*n*=21), two were written for complex interventions, two for devices, and the remaining 17 for general medicine.

### Designing a trial with clustering and learning

#### Choosing a trial design

Eleven out of 28 documents (39%) provided guidance relating to trial design. See D1 in Supplementary Table 1.

The options of trial design depend on the unit of randomisation and the intervention of interest. The key aspects of relevant designs are briefly summarised here. Many design options, and associated limitations, were discussed and no single document provided a single comprehensive summary.

In individually randomised trials, patients are the unit of randomisation [3]. When conducting these trials in surgery, differential expertise between the treatments being investigated can raise issues that can be alleviated by defining eligibility criteria for centres and surgeons, such as years in practice or the number of interventions performed previously [14, 15]. However, applying criteria that are too strict may reduce the generalisability of trial results [16]. Instead, a statistical analysis of inter-rater reliability, between individual centres and surgeons, can provide an understanding of any impact due to expertise. This type of analysis can be useful when considering rolling out the interventions into routine healthcare, see the ‘Analysing a trial with clustering and learning’ section [15].

In cluster randomised trials, groups of patients are the unit of randomisation. These designs are less common and are generally less efficient than individually randomised studies. They require more surgeons and introduce the potential for the treatment comparison to be confounded by the delivery, despite inflating the sample size to account for the intraclass correlation coefficient (ICC) [3, 4, 17].

Expertise-based designs are a half-way house between individual and cluster randomised trials. Patients are individually randomised to surgeon, who treats all patients with a single intervention. This can be the surgeon’s preferred technique or an unavoidable feature in trials comparing interventions delivered by different specialties [4, 14]. This design has the same limitations as cluster trials, and when a surgeon is only performing their preferred technique, shared waiting lists [4] and understanding how the treatment can be rolled out into routine healthcare can be a challenge. Resultantly, this design is relatively uncommon [10, 11].

Tracker designs, proposed by Ergina et al., where new or evolving interventions can theoretically be developed within a single randomised study, and the incremental changes to the intervention tracked within the analysis, would be very challenging in practice [4].

#### Considering who will deliver the intervention

Thirteen out of 28 documents (46%) discussed the importance of deciding who will deliver the intervention. See D2 in Supplementary Table 1.

Some variation in delivery, in part, will depend on the skill and training of those delivering the intervention [4, 14, 18]. As such, the selection of centres and treatment providers was a critical element of design discussed by a number of guidance documents [9, 17, 19, 20]. Any eligibility criteria for participating centres and treatment providers, and a description such as the degree to which they are typical, should be reported [14, 16, 21].

Two guidelines suggested criteria by which recruiting centres should be chosen, such as caseload for the procedure under investigation and ensuring sufficient numbers of the target population [14, 20].

No guidelines provided advice on selecting treatment providers. Treatment providers could be a limited group or all professionals offering the intervention [22]. If it is a limited group, guidance on selecting centres, and reporting requirements, may be looked upon as a proxy for trialists when deciding how to select treatment providers, for example caseload and ensuring specific qualifications [14, 20, 21].

The results of the main trial should report on the number of centres and treatment providers performing each intervention [21].

#### Ensuring that the intervention is standardised

Fifteen out of 28 documents (54%) discussed the importance of standardising the intervention. See D3 in Supplementary Table 1.

Variation in delivery can be reduced by standardising all, or aspects of, the intervention of interest. Limiting variation in treatment delivery may be more desirable in an efficacy trial than a pragmatic, effectiveness study [3, 5]. In pragmatic trials, standardisation might consist of simply informing treatment providers to perform the treatment as usual [14]. Regardless of the stage, trial delivery should be similar at all centres [9] and designed such that a clear description of the procedures performed can be provided [16, 23]. Investigator meetings to prepare investigators and standardise performance were suggested by one document [24].

Monitoring treatment adherence was an important aspect across documents [5, 9, 14, 24, 25]. Suggested methods included reviewing case report forms, videotapes, and audiotapes, extending to decertifying and excluding surgeons not submitting a videotape rated acceptable by an independent committee [14].

Reporting in-depth details of the intervention, and comparator, was required by a number of documents. Aspects required included technical procedures; full details on preoperative, intraoperative, and postoperative care; and the extent to which delivery was permitted to vary between participants, treatment providers, and centres [14, 16, 25].

#### Anticipating changes over time

Eight out of 28 documents (29%) discussed considering changes in the delivery of the intervention over time. See D4 in Supplementary Table 1.

Delivery may still vary irrespective of training, experience, and other steps to enforce standardisation. The amount of variation will depend on the stage and technicality of intervention development [3, 5, 14, 26]. An important aspect of surgical evaluation across the guidelines was that delivery may change over time for pragmatic reasons, changes in external factors, or as a result of expertise developing during the study [3–5].

Expertise can develop over a very long time and so requiring a set expertise level can slow the delivery of surgical trials [4]. Some guidelines discussed evaluating the learning curve within the trial [5] and highlighted this was particularly important in earlier phase trials [26]. In trials comparing more established techniques, the statistical advantages and gain in ‘internal validity’ need to be considered against the loss of generalisability or ‘external validity’ of applying too much emphasis on the learning curve [3].

Reporting learning curve assessment results was required by one document but this was limited to early phase studies [26].

#### Estimating the sample size

Eight out of 28 documents (29%) discussed sample size. See D5 in Supplementary Table 1.

A number of guidance documents highlighted the impact of failing to reduce variation within trial arms by standardising the intervention on the sample size and power calculation, where typical estimates assume that differences between the treatments across centres, or treatment provider, are unbiased estimates of the same quantity [3, 9]. In the presence of multilevel data structures, where variability in individual-level outcomes can reflect higher-level processes, calculations are more complicated [7, 9, 18]. To avoid associated imprecision in results, the sample size should adjust for any clustering effects as estimated by the intraclass correlation coefficient (ICC) and this should be reported in the main result paper [14, 21]. Conversely, two documents that discussed sample size did not comment on adjusting for clusters [15, 20].

#### Ensuring balance of treatment within centre and treatment provider

Six out of 28 documents (21%) discussed ensuring that treatment allocations are equally distributed within centre. See D6 in Supplementary Table 1.

Balancing treatment groups with respect to prognostic factors enhances trial credibility [20, 27]. Ensuring balancing of patients within centre was highlighted as important within many of the guidance documents [9, 20, 27], and similar reasoning would lead surgical trialists to extend this to treatment provider which was not discussed within any document.

Balance can be achieved by stratifying the randomisation and stratifying by centre was a common topic, particularly when centre is expected to be confounded with other prognostic factors [9, 20, 27]. When there are too few patients per centre, stratifying by a larger unit, such as country or region, may be warranted [27]. Despite stratifying by treatment provider not being specifically addressed within the documents, in some circumstances, it may be desirable to stratify for more than just both centre and treatment provider, or treatment provider alone, where numbers allow [27]. The use of more than two stratification factors is rarely necessary [9].

### Analysing a trial with clustering and learning

#### When the randomisation was stratified

Two out of 28 documents (18%) provided guidance on adjusting the analysis following stratification. See A1 in Supplementary Table 1.

Stratifying randomisation and subsequently adjusting the analysis are complementary methods of accounting for prognostic factors, unless the stratification factor was chosen for administrative reasons only [9, 27].

Two documents discussed the issue of adjusting for too many, or too small, strata in the analysis, for which there is no best solution [9, 27]. When included in the randomisation scheme, ignoring centres or adjusting for a large number of small centres might lead to unreliable estimates of the treatment effect and *p*-values [27]. At best, using an unadjusted analysis should be supported by sensitivity analyses that indicate trial conclusions are not affected because of this [27]. As above, the statistical justifications for including centre could be considered to also include treatment provider in surgical trials, but no guidance specifically made this point.

#### When analysing the primary outcome

Two out of 28 documents (18%) provided guidance on adjusting the primary outcome analysis. See A2 in Supplementary Table 1.

Unexplained differences between treatments, for example between adjusted and unadjusted analyses, can jeopardise the trial results [27]. For this reason, when the primary outcome is expected to be influenced by centre or treatment provider, an adjustment should be planned. When the potential value of an adjustment is in doubt, such as little existing prior knowledge, the primary analysis should be an unadjusted analysis, supported by an adjusted analysis [9, 27]. In general, larger datasets generally support more factors than smaller ones and results based on simpler models are generally numerically stable, the assumptions underpinning the statistical model easier to validate and improve generalisability [27].

#### Analysing multi-centre trials

Six out of 28 documents (21%) provided guidance on analysing multi-centre trials. See A3 in Supplementary Table 1.

Investigations into the heterogeneity of the main treatment effect across centre and/or treatment provider were covered by a number of documents [5, 9, 14, 25, 26]. Furthermore, the main trial publication should report methods to adjust for, and results into, clustering by centre or treatment provider [14, 21]. These investigations are critical when a positive treatment effect is found and there are appreciable numbers of subjects per centre [9]. In the simplest multi-centre trial, a single investigator recruits and is responsible for all patients within a single hospital, such that the centre is identified uniquely by hospital. When the definition of a centre is ambiguous, such as a single investigator recruits from several hospitals or a clinical team recruits from numerous clinics, the protocol should provide a definition [9, 25].

Quantitative approaches may comprise a graphical display of the results of individual centres, such as forest plots, or analytical methods, such as a significance test although this generally has low power [9]. One stated that investigations use a model which allows for centre differences but no interaction terms [9]. Fixed or mixed effects models can be used, although mixed models are especially relevant when there is a large number of centres [9, 25].

#### Methods for investigating the learning curve

Four out of 28 documents (14%) provided guidance on analysing the learning curve within centre and/or treatment provider. See A4 in Supplementary Table 1.

Reporting of continuous quality control measures can be useful for all phases of the trial, particularly early phase surgical trials [5, 26]. Time series and longitudinal models or multilevel models can be used to analyse long and short sequences of data respectively [3, 18]. Simpler exploratory methods such as cusum plots enable centres or surgeons to be compared against themselves which can be preferable to surgeons [5, 26].

#### Method for investigating clustering

Five out of 28 documents (18%) provided guidance on investigating clustering due to centre and/or treatment provider. See A5 in Supplementary Table 1.

Hierarchically structured data, such as patients within surgeon, can be analysed using multilevel models or generalised estimating equations (GEEs) [3, 21]. Multilevel models are subject-specific models whereas GEEs are population average. For multilevel models, fixed, random, or mixed effects can be specified to account for clustering [21] and different types of these models allow for flexible data structures [18].

For ordinary linear models, the treatment effect estimate is likely to be similar but not necessarily identical for adjusted and unadjusted models. Adjusted analyses are more efficient, and so a less significant result for unadjusted should not be a concern. For generalised linear or non-linear models, adjusted and unadjusted treatment effects may not have the same interpretation and may provide different results [27].

## Discussion

Trialists should consider the impact of clustering and learning when designing and analysing randomised surgical trials. Considerations should be incorporated into reporting to aid the interpretation and applicability of trial results. This investigation is the first review as to the extent that existing guidance within the UK covers these important effects. Existing guidance documents are identified and summarised, with a focus on aspects relating to clustering effects and the learning curve and their application to surgical trials. Not all documents were written specifically for surgery, yet all contain aspects that can be applied to surgery, for example, the role of the centre in the delivery of treatments in drug trials has some commonalities to the role of the surgeon in delivering a surgical trial. Twice the number of identified documents targeted design aspects than analysis. Whilst a good analysis cannot rescue a poor design, and this may have led to a larger focus on the design on guidance for trialists, there is a notable dearth of analysis guidance available that requires addressing. In addition, there is also scope for guidance on study conduct.

Clustering, at the centre level, was well covered within the design, analysis, and reporting guidance. However, there were inconsistencies with regard to the treatment provider coverage. For example, reporting required that the eligibility of the treatment provider be covered, yet no guidance on the design or analysis covered this [14, 21]. However, this may be due to the original guidance largely not being written specifically for surgery, or indeed complex interventions, where these effects may be more prominent [10, 11]. The role of the centre within conventional drug trials could be extended to provide guidance on the role of the treatment provider in surgery trials [3, 9, 20]. Methods to monitor the quality of delivery of the trial intervention through study conduct have been considered, recommending that stablished protocols that determine prohibited, mandated, and flexible intervention components and monitoring adherence are developed [28]. Yet a specific guidance document that covers the design and analysis of randomised surgical trials, or intervention trials, could address the discrepancies identified within this review to improve the quality of understanding and awareness of these issues [10–12].

A number of guidance documents acknowledged the importance of the surgical learning curve, or delivery changing over time, within design and analysis, particularly in early phase surgical trials or when the interventions differ in their technicality [3–5, 14, 26]. Yet there was little coverage within reporting standards to reflect this, with the surgical learning curve analysis only necessary in the early phase, and not necessarily randomised, trials [26] and broader RCT reporting guidelines only requiring differential expertise be addressed in the discussion [14]. Lack of clear standards, and guidance that is too broad in scope, may lead to reporting how delivery of intervention changes over time, despite its importance, being generally under-recognised in the literature [10, 11].

When designing and analysing a randomised surgical trial, there can be a view that clustering and learning are less pronounced or irrelevant in more pragmatic large-scale trials where the interventions are stabilised and in widespread use. Measures to reduce variation in treatment effects are often introduced into the trial design by defining a minimum level of expertise or providing training for treatment providers [12]. However, despite measures being taken, variation in delivery may remain, and the need to account for the breadth of the setting, learning curve, and experience of surgeons is an area for improvement in more pragmatic RCTs [29–31]. Trialists should therefore be aware of the potential for clustering and learning and routinely consider their impact at the trial outset. Early and careful consideration will improve data collection to ensure that, if required, investigation can be integrated into the planned analysis of the trial. Being able to explore effects will be particularly beneficial if concerns of learning or clustering are raised, or more generally will allow better understanding to contextualise study findings to ultimately support the rollout of the interventions into routine practice.

When reading this review, it is important to consider its limitations. First, country-specific guidance beyond the UK, such as US Food and Drug Administration, was not included. However, international documents that are applicable to other countries, including the UK, were obtained, such as ICH which are followed globally and EMA which are adopted within Europe. Second, only four guidance documents, developed by the same research group, were written specifically for surgery and not written specifically for RCTs, which may explain the lack of specific coverage of the surgeon in the wider set [4, 5, 26, 32]. Third, very little of the guidance documents covered statistical aspects, leaving a trialist to extend the centre-drug connection to surgeon-intervention using existing guidelines [9, 27]. The development of a statistical guidance document that covers randomised surgical trials in more depth would help trialists, in particular statisticians, and the IDEAL framework provide a good basis for this development to be integrated [4, 5, 26, 32].

## Conclusions

This is the first review, to our knowledge, to explore the coverage of guidance for managing clustering effects and the surgical learning curve within the design and analysis of randomised surgical trials. Twice the number of identified documents target design aspects than analysis. Furthermore, no single and complete guidance document exists that covers aspects of learning and clustering leaving trialists to have to access multiple documents to gain full understanding of these considerations.

Existing documents should therefore be extended to incorporate statistical guidance on the management of clustering and learning. The IDEAL framework aligns perfectly with the focus of this work as it is developed specifically for surgical trials and is already widely used by surgical trialists [5]. Future work should address integrating these statistical themes into this framework as a priority. This would encourage better consistency between trialists, improve awareness of these methodological challenges, and support the use of optimal methods within the surgical field.

## Supplementary Information

**Additional file 1: Supplementary Material 1.** List of eligible guidance documents. **Supplementary Material 2.** Additional information on documents obtained by the targeted search. **Supplementary Table 1.** Key criteria coverage across documents summary.

## Data Availability

Datasets used and/or analysed during the current study, which are not already included in this published article, are available from the corresponding author on reasonable request.

## Abbreviations

GEE: Generalised estimating equation
ICC: Intraclass correlation coefficient
RCT: Randomised controlled trial
