# Supplementary material for: Managing clustering effects and learning effects in the design and analysis of randomised surgical trials: a review of existing guidance
Source: Trials. 2022 Oct 11;23:869. doi: 10.1186/s13063-022-06743-6 (PMC9552436; doi:10.1186/s13063-022-06743-6)
Supplement: Supplementary file 1 — Additional file 1: Supplementary Material 1. List of eligible guidance documents. Supplementary Material 2. Additional information on documents obtained by the targeted search. Supplementary Table 1. Key criteria coverage across documents summary. [file 13063_2022_6743_MOESM1_ESM.docx]

**SUPPLEMENTARY MATERIAL**

Supplementary Material 1: List of eligible guidance documents

**The following guidance documents were identified using the EQUATOR Network search engine:**

1. Bilbro NA, Hirst A, Paez A, Vasey B, Pufulete M, Sedrakyan A, McCulloch P; IDEAL Collaboration Reporting Guidelines Working Group. The IDEAL Reporting Guidelines: A Delphi Consensus Statement Stage specific recommendations for reporting the evaluation of surgical innovation. Ann Surg. 2020.
2. Elias KM, Stone AB, McGinigle K, Tankou JI, Scott MJ, Fawcett WJ, Demartines N, Lobo DN, Ljungqvist O, Urman RD; ERAS® Society and ERAS® USA. The Reporting on ERAS Compliance, Outcomes, and Elements Research (RECOvER) Checklist: A Joint Statement by the ERAS® and ERAS® USA Societies. World J Surg. 2019 Jan;43(1):1-8.
3. Gamble C, Krishan A, Stocken D, Lewis S, Juszczak E, Doré C, Williamson PR, Altman DG, Montgomery A, Lim P, Berlin J, Senn S, Day S, Barbachano Y, Loder E. Guidelines for the Content of Statistical Analysis Plans in Clinical Trials. JAMA. 2017;318(23):2337-2343.
4. Jackson DL. Reporting results of latent growth modeling and multilevel modeling analyses: some recommendations for rehabilitation psychology. Rehabil Psychol. 2010;55(3):272-285.
5. de Jager DJ, de Mutsert R, Jager KJ, Zoccali C, Dekker FW. Reporting of interaction. Nephron Clin Pract. 2011;119(2):c158-161.
6. Kalil AC, Mattei J, Florescu DF, Sun J, Kalil RS. Recommendations for the assessment and reporting of multivariable logistic regression in transplantation literature. Am J Transplant. 2010;10(7):1686-1694.
7. Kent DM, Rothwell PM, Ioannidis JP, Altman DG, Hayward RA. Assessing and reporting heterogeneity in treatment effects in clinical trials: a proposal. Trials. 2010;11:85.
8. Lang TA, Altman DG. Basic statistical reporting for articles published in biomedical journals: the "Statistical Analyses and Methods in the Published Literature" or the SAMPL Guidelines. Int J Nurs Stud. 2015;52(1):5-9.
9. van de Schoot R, Sijbrandij M, Winter SD, Depaoli S, Vermunt JK. Guidelines for Reporting on Latent Trajectory Studies (GRoLTS). Structural Equation Modeling: A Multidisciplinary Journal. 2017;24(3):451-467.
10. Schreiber JB. Latent Class Analysis: An example for reporting results. Res Social Adm Pharm. 2016.
11. Vanhie A, Meuleman C, Tomassetti C, Timmerman D, D'Hoore A, Wolthuis A, Van Cleynenbreugel B, Dancet E, Van den Broeck U, Tsaltas J, Renner SP, Ebert AD, Carmona F, Abbott J, Stepniewska A, Taylor H, Saridogan E, Mueller M, Keckstein J, Pluchino N, Janik G, Zupi E, Minelli L, Cooper M, Dunselman G, Koh C, Abrao M, Chapron C, D'Hooghe T. Consensus on Recording Deep Endometriosis Surgery: the CORDES statement. Hum Reprod. 2016 Apr 19. pii: dew067.
12. Wang R, Lagakos SW, Ware JH, Hunter DJ, Drazen JM. Statistics in medicine--reporting of subgroup analyses in clinical trials. N Engl J Med. 2007;357(21):2189-2194.

**The following guidance documents were identified by the targeted search (T)**

*Identified from UK funding bodies (n=2)*

1. Clinical Trials Toolkit –Planning a Randomised Controlled Trial – Points to Consider: Funding proposal National Institute for Health Research web site: National Institute for Health Research; [Available from: <https://www.ct-toolkit.ac.uk/routemap/trial-planning-and-design/downloads/planning-a-randomised-controlled-trial.pdf/>.
2. Craig P, Dieppe P, Macintyre S, Michie S, Nazareth I, Petticrew M. Developing and evaluating complex interventions Medical Research Council web site: Medical Research Council; 2019 [Available from: <https://mrc.ukri.org/documents/pdf/complex-interventions-guidance/>.

*Identified from regulators (n=6)*

1. Committee for Medicinal Products for Human Use (CHMP): Guideline on adjustment for baseline covariates in clinical trials European Medicines Agency Science Medicines Health web site: European Medicines Agency 2015 [Available from: <https://www.ema.europa.eu/en/documents/scientific-guideline/guideline-adjustment-baseline-covariates-clinical-trials_en.pdf>]
2. Group IEW. ICH Harmonised Tripartite Guideline: Structure and Content of Clinical Study Reports E3 ICH Harmonisation for better health web site: International Conference on Harmonisation of Technical Requirements for Registration of Pharmaceuticals for Human Use; 1995 [Available from: <https://database.ich.org/sites/default/files/E3_Guideline.pdf> ]
3. Group IEW. ICH Harmonised Tripartite Guideline: Guideline for Good Clinical Practice E6(R2) ICH Harmonisation for better health web site: International Conference on Harmonisation of Technical Requirements for Registration of Pharmaceuticals for Human Use; 2016 [Available from: <https://database.ich.org/sites/default/files/E6_R2_Addendum.pdf>
4. Group IEW. ICH Harmonised Tripartite Guideline: General Considerations for Clinical Trials E8 ICH Harmonisation for better health web site: International Conference on Harmonisation of Technical Requirements for Registration of Pharmaceuticals for Human Use; 1998 [Available from: <https://www.ema.europa.eu/en/documents/scientific-guideline/ich-e-8-general-considerations-clinical-trials-step-5_en.pdf>
5. ICH Harmonised Tripartite Guideline. Statistical principles for clinical trials. International Conference on Harmonisation E9 Expert Working Group. Stat Med. 1999;18(15):1905-42.
6. Medicines & Healthcare products Medicines Agency (MHRA): Guidance on legislation. Clinical investigations of medical devices – statistical considerations. Medicines & Healthcare products Regulatory Agency 2021. [Available from: [Statistical_considerations_clinical_investigations_-_May_2021.pdf (publishing.service.gov.uk)](https://assets.publishing.service.gov.uk/government/uploads/system/uploads/attachment_data/file/989415/Statistical_considerations_clinical_investigations_-_May_2021.pdf)

*Identified from medical journals (n=8)*

1. Barkun JS, Aronson JK, Feldman LS, Maddern GJ, Strasberg SM, Balliol Collaboration. Evaluation and stages of surgical innovations. Lancet. 2009;374(9695):1089-96.
2. Boutron I, Altman DG, Moher D, Schulz KF, Ravaud P, DJ C, et al. CONSORT Statement for Randomized Trials of Nonpharmacologic Treatments: A 2017 Update and a CONSORT Extension for Nonpharmacologic Trial Abstracts. Annals of Internal Medicine. American College of Physicians; 2017 Jul 4;167(1):40. [PMID: 28630973](https://www.ncbi.nlm.nih.gov/pubmed/28630973)
3. Boutron I, Moher D, Altman DG, Schulz KF, Ravaud P; CONSORT Group. Methods and processes of the CONSORT Group: example of an extension for trials assessing nonpharmacologic treatments. Ann Intern Med. 2008 Feb 19;148(4):W60-6. Doi: 10.7326/0003-4819-148-4-200802190-00008-w1. PMID: 18283201.
4. Campbell MK, Piaggio G, Elbourne DR, Altman DG; for the CONSORT Group. Consort 2010 statement: extension to cluster randomized trials. BMJ. 2012 Sep 4;345:e5661. [PMID: 22951546](http://www.ncbi.nlm.nih.gov/pubmed?term=22951546)
5. Ergina PL, Cook JA, Blazeby JM, Boutron I, Clavien PA, Reeves BC, et al. Challenges in evaluating surgical innovation. Lancet. 2009;374(9695):1097-104.
6. McCulloch P, Altman DG, Campbell WB, Flum DR, Glasziou P, et al. No surgical innovation without evaluation: the IDEAL recommendations. Lancet. 2009;374(9695):1105-12.
7. Schulz K F, Altman D G, Moher D. CONSORT 2010 Statement: updated guidelines for reporting parallel group randomised trials BMJ 2010; 340 :c332 doi:10.1136/bmj.c332
8. Zwarenstein M, Treweek S, Gagnier JJ, Altman DG, Tunis S, Haynes B, Oxman AD, Moher D for the CONSORT and Pragmatic Trials in Healthcare (Practihc) group. Improving the reporting of pragmatic trials: an extension of the CONSORT statement. BMJ 2008; 337;a2390. [PMID: 19001484](http://www.ncbi.nlm.nih.gov/pubmed/19001484)

**Note:** Further information on the relevance of each document obtained from the targeted search is provided in *Supplementary Material 2.*

Supplementary Material 2: Additional information on documents obtained by the targeted search

*UK funding bodies*

Two documents were obtained from UK funding bodies (T1, T2).

T1 was obtained from the NIHR Clinical Trials Toolkit. [*NIHR Toolkit, 2016*] The Toolkit was searched as it provides practical advice to researchers designing and conducting publicly funded clinical trials in the UK, information on best practice and outlines the current legal and practical requirements for conducting clinical trials. Whilst primarily focusing on Clinical Trials of Investigational Medicinal Products (CTIMPs), information and guidance relevant to the wider trial environment is also incorporated, such as T1, which specifically relates to trials where the intervention is a device, therapy or other complex intervention.

T2 was obtained from the Medical Research Council (MRC), which aims to improve human health through world-class medical research by supporting research, including clinical trials, in all disease areas. They work closely with the NHS and UK Health Departments and their guidance on the development, evaluation and implementation of complex interventions which aims to help researchers, funders and users understand methodological and practical constraints in complex intervention evaluation was thus included. [*MRC, 2019*]

*Identified from regulators (n=6)*

Six documents were obtained from regulators of relevance within the UK (T3-T8).

T3 was obtained from the European Medicines Agency (EMA), who supports scientific excellence in the evaluation and supervision of medicines, for the benefit of public health in the European Union. [*EMA, 2015*] They facilitate development and monitor the safety of medicines across their life cycle. T3 is included as discusses adjustment for baseline covariates, particularly important where there are associations between the factor and outcome measure.

T4, T5, T6 and T7 are part of the International Council for Harmonisation of Technical Requirements for Pharmaceuticals for Human Use (ICH) and, due to its specific content, were identified for inclusion in this review. [*ICH E3, 1995; ICH E6, 2016; ICH E8, 1998; ICH E9, 1999*] This series aims to achieve worldwide harmonisation in the development and registration of medicines through guidelines developed via consensus, with regulatory and industry experts working side-by-side.

T8 was obtained from the Medicine & Healthcare products Regulation Agency (MHRA). [*MHRA, 2021*] This executive UK agency, sponsored by the Department of Health and Social Care, was searched as it regulates medicines and medicinal devices within the UK. T2, their document within guidance on legislation, is included as specifically addresses considerations for devices, which require administration from a treatment provider such as surgeon.

*Identified from medical journals (n=8)*

Eight documents were obtained as those endorsed by medical journals (T9-T16).

Three were selected for inclusion as identified from the IDEAL Framework (T9, T13, T14). [*Barkun, 2009; Ergina, 2009; McCulloch, 2009*] The IDEAL framework describes the five stages of surgical therapy innovation: Idea, Development, Exploration, Assessment and Long-term follow-up. Recommendations are provided with respect to study design and reporting in to improve evidence on surgical and interventional therapy innovation. These documents were included due to their clear relevance to the subject matter.

Five were selected as relevant CONSORT documents. (T10-T12, T15, T16) [*Boutron, 2017; Boutron, 2008; Campbell, 2012; Schulz, 2010; Zwarenstein, 2008*] The Consolidated Standards of Reporting Trials (CONSORT) aims to alleviate inadequate reporting of randomised controlled trials. The CONSORT Statement is endorsed by prominent general medical journals, specialty medical journals, and leading editorial organizations. Three extensions, and relevant updates, are included in this guidance summary.

Supplementary Table 1: Key criteria coverage across documents summary

| **Document** | **Key criteria ^A^** | | | | | | | | | | | **Total**  n |
| --- | --- | --- | --- | --- | --- | --- | --- | --- | --- | --- | --- | --- |
|  | **Design** | | | | | | **Analysis** | | | | |  |
|  | D1 | D2 | D3 | D4 | D5 | D6 | A1 | A2 | A3 | A4 | A5 |  |
| *Barkun, 2009* | No | No | Yes | Yes | No | No | No | No | No | No | No | 2 |
| *McCulloch, 2009* | Yes | Yes | Yes | Yes | No | No | No | No | Yes | Yes | No | 6 |
| *Bilbro, 2021* | No | Yes | Yes | Yes | No | No | No | No | Yes | Yes | No | 5 |
| *Boutron, 2008* | Yes | Yes | Yes | Yes | Yes | No | No | No | Yes | No | No | 6 |
| *Boutron, 2017* | Yes | Yes | Yes | Yes | Yes | Yes | No | No | Yes | No | Yes | 8 |
| *Campbell, 2012* | Yes | Yes | No | No | Yes | Yes | No | No | No | No | Yes | 5 |
| *MRC, 2019* | Yes | No | Yes | Yes | Yes | No | No | No | No | Yes | Yes | 6 |
| *Elias, 2019* | No | Yes | Yes | No | No | No | No | No | No | No | No | 2 |
| *Ergina, 2009* | Yes | Yes | Yes | Yes | No | No | No | No | No | No | No | 4 |
| *EMA, 2015* | No | No | No | No | No | Yes | Yes | Yes | No | No | Yes | 4 |
| *ICH E3, 1995* | No | No | Yes | No | No | Yes | No | No | Yes | No | No | 3 |
| *ICH E6, 2016* | No | Yes | No | No | No | No | No | No | No | No | No | 1 |

Supplementary Table 1: Key criteria coverage across documents summary

| **Document** | **Key criteria ^A^** | | | | | | | | | | | **Total**  n |
| --- | --- | --- | --- | --- | --- | --- | --- | --- | --- | --- | --- | --- |
|  | **Design** | | | | | | **Analysis** | | | | |  |
|  | D1 | D2 | D3 | D4 | D5 | D6 | A1 | A2 | A3 | A4 | A5 |  |
| *ICH E8, 1998* | No | No | Yes | Yes | No | No | No | No | No | No | No | 2 |
| *ICH E9, 1999* | Yes | Yes | Yes | No | Yes | Yes | Yes | Yes | Yes | No | No | 8 |
| *Jackson, 2010* | Yes | Yes | No | No | Yes | No | No | No | No | Yes | Yes | 5 |
| *Jager, 2011* | No | No | No | No | No | No | No | No | No | No | No | 0 |
| *Gamble, 2017* | No | No | No | No | No | No | No | No | No | No | No | 0 |
| *Kalil, 2010* | No | No | No | No | No | No | No | No | No | No | No | 0 |
| *Kent, 2010* | No | No | No | No | No | No | No | No | No | No | No | 0 |
| *Lang, 2015* | No | No | No | No | No | No | No | No | No | No | No | 0 |
| *MHRA, 2021* | Yes | Yes | No | No | Yes | Yes | No | No | No | No | No | 4 |
| *NIHR Toolkit, 2016* | No | Yes | Yes | No | Yes | No | No | No | No | No | No | 3 |
| *Schoot, 2017* | No | No | No | No | No | No | No | No | No | No | No | 0 |
| *Schreiber, 2016* | No | No | No | No | No | No | No | No | No | No | No | 0 |

Supplementary Table 1: Key criteria coverage across documents summary

| **Document** | **Key criteria ^A^** | | | | | | | | | | | **Total**  n |
| --- | --- | --- | --- | --- | --- | --- | --- | --- | --- | --- | --- | --- |
|  | **Design** | | | | | | **Analysis** | | | | |  |
|  | D1 | D2 | D3 | D4 | D5 | D6 | A1 | A2 | A3 | A4 | A5 |  |
| *Schultz, 2011* | Yes | No | Yes | No | No | No | No | No | No | No | No | 2 |
| *Vanhie et al, 2016* | No | No | Yes | No | No | No | No | No | No | No | No | 1 |
| *Wang, 2007* | No | No | No | No | No | No | No | No | No | No | No | 0 |
| *Zwarenstein, 2008* | Yes | Yes | Yes | No | No | No | No | No | No | No | No | 3 |
| **Total**  n (n/28%) | 11 (39%) | 13 (46%) | 15 (54%) | 8  (29%) | 8  (29%) | 6  (21%) | 2  (7%) | 2  (7%) | 6  (21%) | 4  (14%) | 5  (18%) |  |

^A^ **Key criteria:** D1: Choosing a trial design; D2: Considering who will deliver the intervention; D3: Ensuring that the intervention is standardised; D4: Anticipating changes in delivery over time; D5: Estimating the sample size; D6: Ensuring balance of treatment within centre and treatment provider; A1: When the randomization was stratified; A2: When analyzing the primary outcome; A3: Analysing multicenter trials; A4: Methods for investigating the learning curve; A5: Methods for investigating clustering.
